# The Oligopeptide Permease Opp Mediates Illicit Transport of the Bacterial P-site Decoding Inhibitor GE81112 †

**DOI:** 10.3390/antibiotics5020017

**Published:** 2016-05-24

**Authors:** Alessandro Maio, Letizia Brandi, Stefano Donadio, Claudio O. Gualerzi

**Affiliations:** 1 Laboratory of Genetics, University of Camerino, via Gentile III da Varano, Camerino 62032 (MC), Italy; Alessandro.Maio@ppdi.com (A.M.); letizia.brandi@unicam.it (L.B.); 2 NAICONS Scrl, Viale Ortles 22/4, Milano 20139, Italy; s.donadio@naicons.com

**Keywords:** peptide antibiotic, MIC, translation inhibitors, peptide transport, antibiotic resistance, cross resistance

## Abstract

GE81112 is a tetrapeptide antibiotic that binds to the 30S ribosomal subunit and specifically inhibits P-site decoding of the mRNA initiation codon by the fMet-tRNA anticodon. GE81112 displays excellent microbiological activity against some Gram-positive and Gram-negative bacteria in both minimal and complete, chemically defined, broth, but is essentially inactive in complete complex media. This is due to the presence of peptides that compete with the antibiotic for the oligopeptide permease system (Opp) responsible for its illicit transport into the bacterial cells as demonstrated in the cases of *Escherichia coli* and *Bacillus subtilis*. Mutations that inactivate the Opp system and confer GE81112 resistance arise spontaneously with a frequency of *ca.* 1 × 10^−6^, similar to that of the mutants resistant to tri-l-ornithine, a known Opp substrate. On the contrary, cells expressing extrachromosomal copies of the *opp* genes are extremely sensitive to GE81112 in rich medium and GE81112-resistant mutations affecting the molecular target of the antibiotic were not detected upon examining >10^9^ cells of this type. However, some mutations introduced in the 16S rRNA to confer kasugamycin resistance were found to reduce the sensitivity of the cells to GE81112.

## 1. Introduction

The spread of multiple antibiotic resistant bacteria and of life threatening “super-bugs” calls for a massive effort by the scientific community towards the development of novel antibacterial molecules that may help mankind to cope with the present “antibiotic emergency” [1].

**Figure 1:**
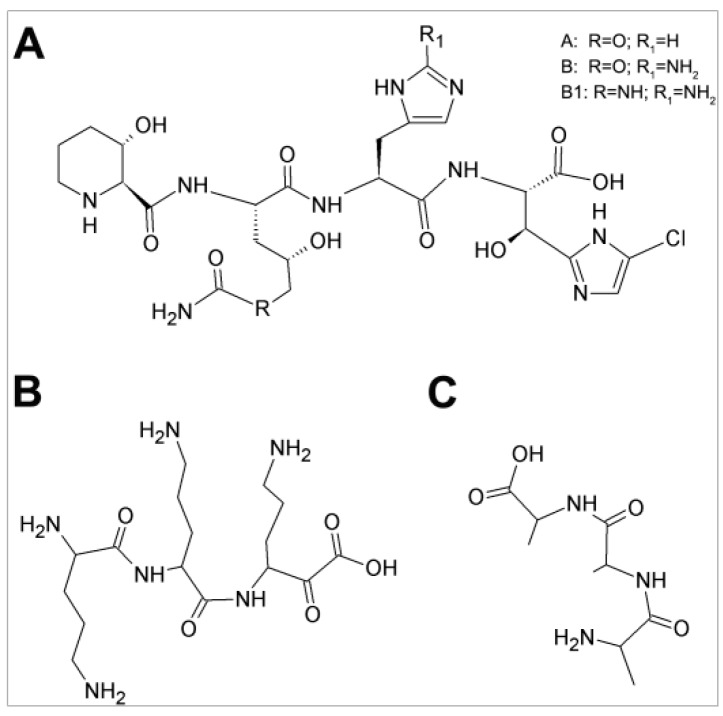
Structures of GE81112, tri-ornithine and tri-l-alanine: (**A**) GE81112 (variant B, MW = 658 Da), the chlorine-containing tetrapeptide antibiotic consists of four non-proteinogenic amino acids (3-hydroxypipecolic acid, 2-amino-5-[(aminocarbonyl) oxy]-4-hydroxypentanoic acid, histidine, and 5-chloro-2-imidazolylserine) [2,4]; (**B**) tri-l-ornithine; and (**C**) tri-l-alanine.

GE81112 is a highly hydrophilic chlorine-containing, non-cyclic, “non-ribosomally” synthesized tetrapeptide belonging to a structurally novel class of antibiotics. It is constituted by four non-proteinogenic l-amino acids; throughout this work variant B (658 Da), the most active of the three structural variants of this antibiotic was used (Figure 1A) [2,3,4]. Fourteen biosynthetic genes (*get*A–N) involved in the biosynthesis of this antibiotic have been identified within a larger biosynthetic gene cluster within the producer *Streptomyces* sp. l-49973 strain; these genes have been cloned, sequenced and partially characterized [4].

GE81112 exclusively inhibits bacterial protein synthesis interfering with an underexploited target within the translational apparatus, namely the binding of initiator fMet-tRNA to the 30S subunit [3,5]. Although the target of GE81112 is superficially similar to that of Furvina^®^ [6], biochemical and structural biology data show that the mechanism of action of these two antibiotics is different. In fact, unlike Furvina^®^ that prevents the initial ribosomal binding of the initiator tRNA [6] to produce a 30S pre-initiation complex [7], GE81112 prevents the subsequent first order isomerization of the 30S pre-initiation complex that upon codon-anticodon interaction in the P-site yields a “locked” 30S initiation complex [5,7]. In particular, GE81112 was shown to bind to the P-site of the 30S subunit and to stabilize the anticodon stem loop of the initiator tRNA in a distorted conformation so as to prevent P-site decoding and stalling 30S subunit in the unlocked 30S pre-*IC* state [5].

Overall, the existing data seem to indicate that GE81112 could be a promising pharmacophore from which one could derive a new class of anti-infectives for which, to the best of our knowledge, no resistance has yet developed in nature.

In light of this, the aim of this study was to investigate the microbiological activity of GE81112 as a function of the growth media of the target bacteria and the mechanism by which this antibiotic enters the cells. Furthermore, the nature of mutations conferring resistance to GE81112 was investigated.

## 2. Results

An important property to be considered when a new molecule is scrutinized for its possible development into an effective antibiotic is its bacteriostatic and/or bactericidal efficacy as well as its specificity and its spectrum of action. The natural tetrapeptide GE81112 is endowed with a potent and selective inhibitory activity against bacterial translation due to its interference with a totally unexploited antibiotic target.

However, when the microbiological activity of GE81112 was tested with a panel of microorganisms under different growth conditions, rather puzzling results were obtained (Table 1). As seen from the table, the antibiotic proved to be fairly effective (<10 μg/mL Minimal Inhibitory Concentration (MIC)) in rich media against some Gram-positives such as two clinical isolates of *Staphylococcus haemolyticus* (one sensitive and one resistant to methicillin) and against a clinical isolate of the Gram-negative *Moraxella catarrhalis*. However, GE81112 was totally ineffective (MIC > 500 μg/mL) against other Gram-positive (*S. aureus*, *Streptococcus pyogenes*, and *Bacillus subtilis*) and Gram-negative (*Escherichia coli, Haemophilus influenza*, and *Pseudomonas aeruginosa*) bacteria, despite the fact that these are sometimes closely related, belonging to the same *Staphylococcus* genus or *Pseudomonadales* order (*i.e.*, *M. catarrhalis* and *P. aeruginosa*). What is remarkable is that, with the exception of *P. aeruginosa,* the same bacteria (*S.* *aureus*, *B. subtilis*, and *E. coli*) that are insensitive to GE81112 in complete medium are instead sensitive when grown in minimal or in chemically defined rich media.

**Figure 2:**
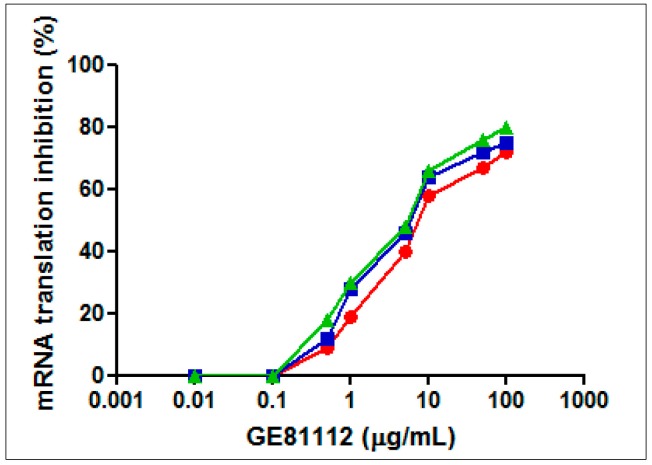
Effect of GE81112 on *in vitro* mRNA translation. Translation of 027IF2Cp(A) mRNA was carried out with cell-free extracts (S30 fractions) prepared from *E. coli* MRE600 (green triangles), *E. coli* DH5α (blue squares) and *P. aeruginosa* 1156 (red diamonds) in the presence of GE81112 in the amounts indicated in the abscissa. The conditions for mRNA translation are described in Material and Methods. *P. aeruginosa* 1156 is a clinical isolate resistant to chloramphenicol, clindamycin, erythromycin, streptogramin, fusidic acid, kanamycin, lincomycin, tetracycline, gentamycin, and streptomycin. One hundred percent activity corresponds to 250.2, 213.7, and 237.3 pmol phenylalanine incorporated in the S30 systems of *E. coli* MRE600, *E. coli* DH5α and *P. aeruginosa* 1156, respectively.

A possible explanation for these findings could be a different efficiency by which GE81112 reaches the 30S ribosomal subunits that represent its target within the cells. In fact, *in vitro* mRNA translation was shown to be inhibited equally well by GE81112 in cell-free extracts prepared from bacteria that are sensitive to the antibiotic only in minimal media (e.g., *E. coli*) or in neither rich nor poor medium (e.g., *P. aeruginosa*), although in the latter case the half maximal inhibitory concentration (IC_50_) is somewhat higher (Figure 2).

Thus, it can be hypothesized that the presence of some inhibitory/inactivating molecule in the rich media is the cause of the strikingly different antibiotic sensitivity in minimal and rich media displayed by the same bacteria. In the particular case of *P. aeruginosa*, it must be surmised that GE81112 always fails to enter in these cells and/or that it is ejected from them by an efficient multidrug resistance efflux pump such as MexAB-OprM or MexCD-OprJ [8,9]. Of course, an alternative explanation could be that the *in vivo* target of GE81112 is not the translational machinery; it could instead be a biosynthetic pathway required for the production of an essential component such as a vitamin, an amino acid, *etc.* This explanation is unlikely in light of the evidence that *in vivo* GE81112 inhibits radioactive methionine incorporation into an acid-insoluble product [3]. Nevertheless, this possibility was investigated by comparing the antimicrobial activity of GE81112 towards *E. coli* and *S. aureus* in a “crude” rich medium and in a complete medium having a chemically defined composition. As seen in Table 1, both *E. coli* and *S. aureus* are not affected by GE81112 in MH broth but become rather sensitive to this antibiotic (MIC = 2–4 μg/mL) in a complete, chemically defined medium. This finding excludes the possibility that the GE81112 target is a biosynthetic pathway. Furthermore, the antimicrobial activity of GE81112 is only slightly reduced in chemically defined complete medium compared to minimal medium. This finding indicates that the ineffectiveness of the antibiotic in complete medium is not due to the concentration of nutrients.

The different activity of GE81112 in different growth media could be due to the presence, only in the “crude” rich media, of inhibitory molecules or of proteins that could sequester or inactivate the antibiotic, thereby reducing its efficacy. This possibility was tested by measuring the anti-bacterial efficacy of GE81112 in minimal media supplemented or not with standard protein molecules or with their proteolytic hydrolysates. The results presented in Table 2 indicate that, whereas bovine serum albumin (BSA), fetal bovine serum (FBS) and casein had only marginal effects on the microbiological activity of GE81112, the hydrolysates of casein and BSA reduced considerably the antibacterial activity of the antibiotic.

**Figure 3:**
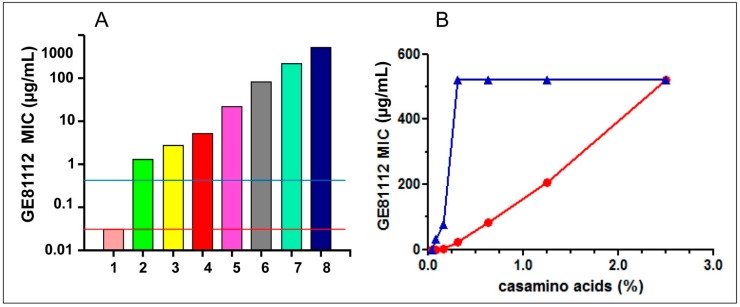
Effect of various additions to Minimal media on the antibacterial activity of GE81112: (**A**) The MIC of GE81112 was tested on *E. coli* MG1655 growing in David Mingioli minimal medium supplemented with the following % concentrations of casamino acids: from 1 through 8 = 0; 0.04; 0.08; 0.16; 0.31; 0.63; 1.25; and 2.50. The horizontal lines indicate the MIC measured in the presence of 0.1 mg/mL of individual amino acids (red line) and 1.25% pooled purified amino acids (blue line). The microtiter wells were filled with 100 μL medium inoculated with 10^4^ cfu/mL and the MICs (expressed as μg/mL) determined after 24 h growth at 37 °C. (**B**) Effect of increasing casamino acids concentrations (indicated in the abscissa) on the MIC of GE81112 on *E. coli* MG1655 (red circles) and *Bacillus subtilis* ATCC6633 (blue triangles). The experimental conditions are the same as indicated in panel A.

These data indicate that the low level of GE81112 activity in rich media is not caused by its non-specific adsorption to a protein, but is due instead to some molecule(s) present in the protein hydrolysates. Thus, molecules likely present in the hydrolysates were individually tested for their ability to interfere with the antibacterial activity of GE81112. As seen in Figure 3A, pure amino acids, added individually or in a pool to minimal medium did not significantly diminish the anti-microbial activity of the antibiotic. Likewise, the addition of vitamins and nitrogen-containing bases had no effect, whereas the addition of casamino acids (between 0.15% and 0.4%) resulted in a dramatic, dose-dependent increase of the MIC value of GE81112 for both *E. coli* and *B. subtilis* (Figure 3B).

The possible existence of species-specific differences in the extent to which different bacteria might be protected by molecules present in rich media is illustrated by the finding that in *B. subtilis* the adverse effect on the MIC of GE81112 manifests itself at ~4-fold lower concentrations of casamino acids than in *E. coli* (Figure 3B). In turn, such differences could account for some of the differences in the MIC of GE81112 detected for bacteria belonging to the same *Streptococcus* genus (e.g., *S. pyogenes vs.* *S. pneumoniae*) (Table 1).

A potentially relevant difference between the individual amino acids, whose additions do not influence GE81112 activity, and casamino acids and protein hydrolysates that cause a sharp increase of the MIC value could be the presence of di-, tri- and oligopeptides that may compete with the antibiotic for an active import system, provided that the tetrapeptide GE81112 is actively pumped into the cell by one of the bacterial peptide transporting systems. Indeed, several peptide transport systems exist in *E. coli* as well in *B. subtilis* and the peptides actively transported inside the cells can support the growth of bacteria auxotrophic for amino acids and/or play other roles (see Discussion). The existence of transport systems that may introduce antibiotics into the bacterial cell is also substantiated by the fact that toxic peptides such as tri-l-ornithine (Figure 1B), bialaphos, biphenomycin, phaselotoxin, *etc.* can be actively transported inside the cell causing antibacterial effects [10].

The tetrapeptide nature of GE81112 and the substrate specificity of these transport systems seemed to rule out the involvement of dipeptide and tripeptide permease implicated in dipeptide and tripeptide transport, respectively, and pointed instead to the Opp transport system that is capable of importing larger oligopeptides inside the bacterial cell without selectivity for size, composition, sequence or charge.

Opp belongs to the ATP-binding cassette (ABC) transporter superfamily and has been identified in several gram-positive and gram-negative bacteria [11,12,13], including *P. aeruginosa* [14] and is responsible for the entry of tri-l-ornithine inside the cells. As seen in Table 1, the antimicrobial activity of GE81112 *vis-à-vis* both *E. coli* and *B. subtilis* in minimal media is influenced by the size of the inoculum and when the cfu/mL is increased from 10^4^ to 10^6^, the MIC is increased 15–30-fold; employing larger inocula it is possible to see some microtiter wells with normal or near-normal growth (“skips“) within the inhibitory concentrations range. These skips are due to spontaneous GE81112-resistant mutants appearing with a frequency of approximately 1 × 10^−6^ in both *E. coli* MG1655 and *B. subtilis* ATCC6633. This frequency is compatible with mutations causing the loss of a complex function rather than altering a hypothetical target such as 16S rRNA and similar to that of the tri-l-ornithine-resistant mutations. In fact, mutants resistant to tri-l-ornithine due to the loss of the oligopeptide permease (Opp)-mediated active transport system arise with similar frequencies (*i.e.* 7 × 10^−6^–1.6 × 10^−5^). The similar rate at which GE81112- and tri-l-ornithine-resistant mutations arise represents a clue that GE81112 and tri-l-ornithine may enter the cells via the same route. A further confirmation of this premise comes from our finding that bacterial cells display cross-resistance to l-tri-ornithine and GE81112. In fact, upon plating *E. coli* MG1655 on minimal medium agar plates, we were able to isolate 100 colonies resistant to tri-l-ornithine and 100 colonies resistant to GE81112. Further analysis demonstrated that all 100 tri-l-ornithine-resistant mutants and all 100 GE81112-resistant mutants that were isolated displayed cross resistance to GE81112 (10 μg/mL) and to tri-l-ornithine (100 μg/mL), respectively. This finding provides a strong indication that the mechanism leading to tri-l-ornithine resistance is the same that causes GE81112 resistance. Furthermore, although no attempt was made to clarify the nature of the mutations causing the double resistance, it seems safe to hypothesize that the mutants that we have isolated were affected in the Opp-mediated peptide transport. This in light of the fact that all tri-l-ornithine resistant mutants previously isolated have proven to be *opp^−^* mutants and that mutations within the *opp* locus occur at a frequency which is at least two orders of magnitude higher than the average mutation rate in *E. coli* [15].

Although early studies concluded that tri-l-ornithine inhibits translation *in vivo*, this molecule, unlike GE81112, was shown to be unable to inhibit mRNA translation in cell-free extracts *in vitro* [16]. Therefore, the most likely explanation for the observed tri-l-ornithine/GE81112 cross-resistance is that the mutations have inactivated the same transport mechanism that allows these two molecules to enter the cell.

**Figure 4:**
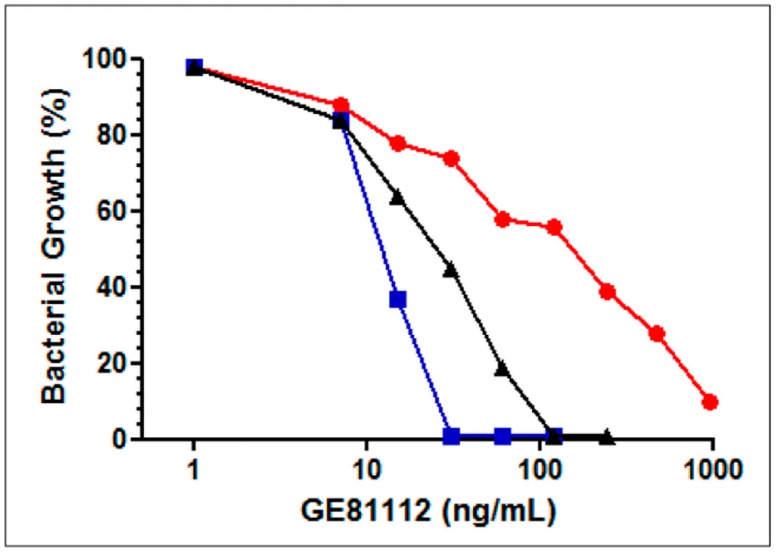
Effect of tri-l-alanine and l-leucine on the antibacterial activity of GE81112. Effect of the GE81112 concentrations indicated in the abscissa on the growth in David Mingioli minimal medium of *E. coli* MG1655 in the absence of other additions (black triangles), in the presence of 25 μg/mL tri-l-alanine (red circles), of 100 μg/mL L-leucine (blue squares). The microtiter wells were filled with 100 μL medium inoculated with 10^4^ cfu/mL and the bacterial growth determined from the A_620_ attained after 24 h at 37 °C.

To test the hypothesis that Opp is indeed responsible for an active transport of GE81112 into the *E. coli* cells, the antibacterial activity of this antibiotic was assayed in the presence of tri-l-alanine and l-leucine. It can be seen that the inhibitory activity of GE81112 decreases in the presence of tri-l-alanine, a known non-toxic Opp substrate that likely competes with GE81112 for this pump [17]. A parallel experiment showed that the inhibitory activity of the antibiotic is potentiated by L-leucine (Figure 4). The explanation for the latter finding is that the presence of l-leucine induces *opp* expression and reduces the level of the transcriptional factor Lrp that exerts a negative control on the Opp-mediated entry of oligopeptides into *E. coli* [17,18]. Thus, taken together, the results of Figure 4 support the notion that GE81112 is transported by Opp.

To obtain conclusive evidence that Opp is responsible for the illicit transport of GE81112 inside the cells, the effect of this antibiotic was investigated on *E. coli 5012* and on its isogenic *E. coli SS320* strain [18]. In *E. coli 5012*, the chromosomal region comprising the *opp* operon is deleted resulting in an *opp^−^* mutant that, unlike the isogenic wt strain, is insensitive to tri-l-ornithine (Table 3). Furthermore, as seen from the results reported in this table, the growth of *E. coli 5012* on minimal medium agar plates is not inhibited by GE81112, whereas the agar plates of the isogenic wt strain *E. coli SS320* show a large growth inhibition halo, much larger than that produced by tri-l-ornithine.

To obtain additional evidence for the involvement of Opp in the transport of GE81112, *E. coli 5012* cells were transformed with pBΦ30 (carrying *oppA*) and/or pB2 (carrying *oppBCDF*) [19] and tested for their sensitivity to tri-l-ornithine and GE81112. As seen from Table 3, the expression *in trans* of *oppA* together with *oppBCDF* in an *E. coli* SS5012 background causes these transformants to become sensitive to both GE81112 and tri-l-ornithine, whereas the expression of either *oppA* or *oppBCDF* alone produces no inhibitory effect. Finally, the wt *E. coli SS320* becomes more sensitive to growth inhibition by both GE81112 and tri-l-ornithine after transformation with both pBΦ30 and pB2.

Taken together, these data indicate that in *E. coli*: (i) Opp is responsible for the illicit transport of both GE81112 and tri-l-ornithine; (ii) deletion of the *opp* operon confers resistance to these two inhibitors; (iii) the entire *opp* operon is necessary for the illicit transport of the oligopeptides; and (iv) the presence of extra copies of the operon in an *opp* wt strain can increase its susceptibility to both GE81112 and tri-l-ornithine.

The analysis of the antibacterial efficiency of GE81112 yielded some puzzling results insofar as bacteria with some obvious kinship displayed different sensitivity to the antibiotic even in minimal media. A striking example of this phenomenon is the fact that some strains of *E. coli* such as MG1655 (MIC = 0.06 μg/μL see Table 1) and MRE600 (MIC~0.1 μg/μL) were found to be sensitive, whereas other strains such as JM109 and DH5α were not, both displaying a MIC > 350 μg/μL. Having established that the sensitivity to GE81112 depends primarily upon the presence of an active Opp transport system, we reasoned that because the *opp* genes are not essential for the bacterial growth in rich, complete media, perhaps extensive growth in the laboratory under optimal nutritional conditions may have caused the accumulation of Opp-inactivating mutations in some bacterial strains. That this might indeed be the case is shown by the results presented in Table 4. It can be seen that the growth of *E. coli* MG1655 is inhibited by GE81112 and tri-l-ornithine, whereas that of *E. coli* DH5α remains unaffected. Since the DH5α strain derives from MG1655, it is likely that mutation(s) within the *opp* gene sequence of the former strain could account for the different susceptibility to GE81112 inhibition. However, when *E. coli* DH5α is transformed with plasmids carrying *oppA*, *oppBCDF* or both, the sensitivity to these two molecules is restored. The Opp pump is constituted by a complex of five proteins. Among these, OppA is responsible for peptide binding, whereas OppBCDF constitute the core domain of the permease. In particular, the hydrophobic transmembrane OppB and OppC domains are predicted to form a pore with 12 transmembrane segments required for the transport of oligopeptides substrates [13]. It is somewhat surprising that in the case of the DH5α strain, unlike with the SS5012 *(ΔoppABCDF)* mutant shown above (Table 3), the sensitivity to GE81112 and to tri-l-ornithine can be restored by the expression *in trans* of either *oppA* or *oppBCDF* alone (Table 4). This behavior may have something to do with the special nature of the mutation that renders the DH5α strain insensitive to the inhibition. The observed suppression of the resistance phenotype could be due to the expression of a wt OppA in one case and to the formation of additional pores in the bacterial membrane by OppBCDF in the other. Alternatively, it is also possible to hypothesize that the resistance to toxic peptides displayed by the DH5α strain is due a failure to assemble correctly the permease complex and that overexpression of either OppA or OppBCDF may suppress this phenotype.

As mentioned above, GE81112-resistant mutants arise at a fairly high frequency as a result of Opp inactivation. The availability of *E. coli* cells expressing multiple copies of the *opp* operon allowed us to bypass these mutations and search instead for mutants that have acquired GE81112 resistance as a result of other types of modifications, possibly affecting the molecular target of the antibiotic. However, no resistant mutants were detected after screening >10^9^ colonies. This finding indicates that GE81112-resistant mutants do not arise spontaneously with high frequency in loci other than *opp*.

The ribosomal localization of GE81112, as determined by a recent crystallographic study [5], places this antibiotic in a position that is not too far away from the binding site of kasugamycin [20,21]. For this reason, some ribosomal mutants bearing 16S rRNA base substitutions that cause kasugamycin resistance [20,21] were tested for their sensitivity to GE81112 inhibition. As seen from Table 5, when *in vitro* mRNA translation was carried out with extracts derived from cells bearing mutations of two bases (A794 and G926) that strongly reduce the inhibitory power of kasugamycin, we detected also an increase of the IC_50_ of GE81112, although the effect is not as dramatic as in the case of kasugamycin. Finally, mutation of A1518 was found to affect exclusively the inhibition by kasugamycin.

The binding site of kasugamycin on the 30S subunit has been elucidated at atomic resolution and both A794 and G926 are shown to be hydrogen bonded with the antibiotic [20,21]. In the case of GE81112, although the position of this molecule on the 30S subunit has not been determined with the same high level of resolution [5], it is nevertheless possible to say that this antibiotic does not establish a direct contact with the same bases. This would indicate that the GE81112 resistance results mainly from conformational effects of the mutations. This could also explain the lower level of resistance that these changes cause on GE81112 with respect to kasugamycin.

As seen above, like *E. coli,* *B. subtilis* is also insensitive to GE81112 inhibition in rich complex media but sensitive in minimal medium, the inhibition being reversed by the addition of casamino acids (Figure 3B). Despite this superficial similarity between *E. coli* and *B. subtilis*, in light of the fact that two oligopeptide transport systems (Opp and App) are present in the latter Gram-positive bacterium and because our previous MIC analyses could not distinguish between Opp- and App-dependent transport of GE81112, it seemed important to check whether the mechanism by which GE81112 enters the *B. subtilis* cells is the same as that found to operate in *E. coli*.

For this purpose, we compared the sensitivity of *B. subtilis* to GE81112 and to bialaphos, the latter being a toxic tripeptide that is actively transported by Opp in this microorganism where it is hydrolyzed to produce phosphinothricin that in turn inhibits glutamine synthetase [10].

Ten independent bialaphos resistant clones were isolated and analyzed for their sensitivity to GE81112 and none of them was found to be inhibited by this antibiotic. Likewise, none of the 10 clones that we isolated as being resistant to GE81112 proved to be sensitive to bialaphos. In light of the different nature of the inhibition target of GE81112 (the P-site of the 30S subunit) and bialaphos (glutamine synthetase), the absence of cross-resistance indicates that the inactivation of the same transport mechanism is responsible for the resistance.

**Figure 5:**
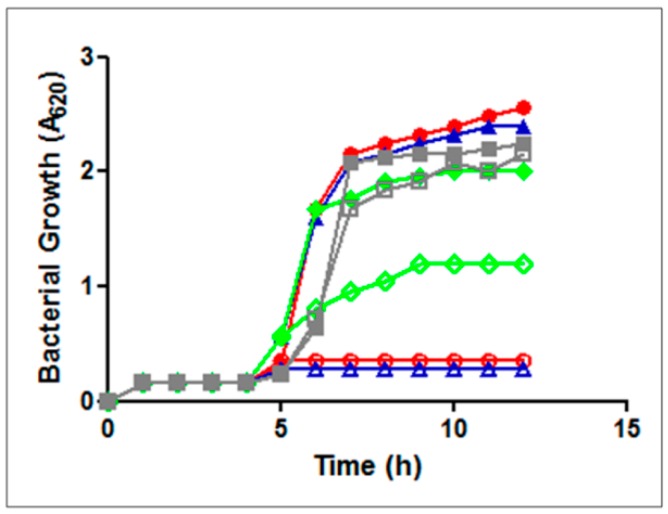
Growth of *B. subtilis* strains in minimal medium in the absence (solid symbols) or in the presence (open symbols) of 2μg of GE81112 added 4 h after inoculation. *B. subtilis* strains were: JH14115 opp^+^app^+^ (blue); JH642 opp^+^app^−^ (red); JH14116 opp^−^app^+^ (green); and JH14115 opp^−^app^−^ (gray).

Evidence that the transport of GE81112 is mainly carried out by Opp and only marginally by App was obtained by comparing the growth of isogenic *B. subtilis* strains (opp^+^app^+^, opp^+^app^−^, opp^−^app^+^ and opp^−^app^−^) [22,23] in the presence of GE81112. As seen from Figure 5, no growth inhibition is observed in the strain lacking both Opp and App pumps (strain JH12795) whereas complete inhibition occurs when the cells have a functional Opp (strains JH642 and JH14115), regardless of App. When App is active in the absence of the Opp function (strain JH14116) only a limited inhibition of bacterial growth is observed. Furthermore, since *app* is expressed in *B. subtilis* only at the end of the exponential growth [24], it seems clear that the role of App in the active transport of GE81112 is restricted to within a limited time window.

## 3. Discussion

GE81112 is a powerful inhibitor of the early step of protein synthesis, namely the P-site decoding of the initiation triplet by the initiator fMet-tRNA that accompanies the transition from a 30S pre-initiation complex to a locked 30S initiation complex [3,5].

The central observation described in this work is that the natural tetrapeptide antibiotic GE81112 enters inside both Gram-positive and Gram-negative bacteria by an illicit active transport mediated by the oligopeptide permease Opp. Many types of peptides are actively transported into the bacterial cells by oligopeptide permease (Opp) to serve different functions. In many cases the imported peptides can be a source of carbon and nitrogen but in other cases they could serve more complex functions. For instance, in *Streptococcus thermophilus* the oligopeptide transport system is essential for the development of natural competence [25] and the Phr peptides of *B. subtilis* regulate the development of environmentally resistant spores and the ability to take up exogenous DNA (genetic competence) [26], whereas the mating pheromones of *Enterococcus faecalis* regulate cell–cell transfer of plasmids [27]. Opp is conserved in many bacteria [28] and, being a non-specific transporter, it is not surprising that it could be involved in the illicit transport [11] of bacterial-toxic peptides such as tri-l-ornithine or, as in the case described here, a peptide antibiotic such as GE81112.

At variance with the strong inhibition displayed *in vitro* by this antibiotic on the cell free systems derived from all bacterial species tested so far, the anti-microbiological activity of GE81112 is not particularly efficient, especially on bacteria growing in rich media.

Nevertheless, in light of the increasing number of infections caused by multi-resistant strains of *Staphylococci,* it is particularly relevant that a clinical isolate of a methicillin resistant *S. haemolyticus* is sensitive to GE81112, even in complete medium and that *S. aureus* is sensitive to GE81112, at least in complete, chemically defined medium. Ultimately, the latter pathogen may also turn out to be a good target of GE81112 inhibition because this bacterium contains at least two *opp* operons essential for its survival in different infection environments [29].

The observation that different bacterial species are differently affected by GE81112 inhibition does not depend upon a different affinity of the highly conserved ribosomal target for the antibiotic, but can be instead explained by the different efficiency by which Opp ensures the entry of the antibiotic inside the cells. Accordingly, the poor microbiological activity of GE81112 towards bacteria growing in complete, rich media was shown to be due to the presence of peptides that give rise to a harsh competition with GE81112; however, we showed that the presence of extra copies of Opp enable the cells to become more GE81112-sensitive in minimal medium and also very sensitive in complete media.

In light of this finding, it would be particularly interesting to check if and under which conditions *Borrelia burgdoferi*, the causative agent of the Lyme disease, is sensitive to GE81112 inhibition. The genome of this spirochete is deficient in biosynthetic genes involved in fatty acids, nucleic acids, and amino acids biosynthesis but contains five copies of *oppA* [30]. Thus, to overcome its auxotrophy for several amino acids *B. burgdorferi* imports oligopeptides via an Opp-mediated transport [31,32].

In agreement with the finding that the Opp oligopermease system acts like a Trojan horse by illicitly introducing GE81112 inside the bacterial cells, all the GE81112-resistant mutants that spontaneously arise at a fairly high frequency turned out to have acquired Opp-inactivating mutations. However, it is noteworthy that no GE81112-resistant mutants could be detected when the antibiotic internalization defect is bypassed by the presence of multiple copies of the *opp* operon. This finding indicates that mutations altering the antibiotic target are not a common occurrence or if they occur, that they have a serious fitness cost. This seems to be a promising finding in the expected event that the tetrapeptide molecule can be chemically modified so as to enter the bacterial cells without the need for the Opp transport.

## 4. Materials and Methods

### 4.1. Bacterial Strains and Plasmids

The bacterial strains used throughout this work as well as their genetic characteristics and origin are listed in Table 6**.** The plasmids used are listed in Table 7.

### 4.2. Antimicrobial Activity

The antimicrobial activity of the antibiotics was determined as Minimal Inhibitory Concentration (MIC) against the microorganisms listed in the tables. The MIC was determined as the minimal concentration capable of preventing a visible bacterial growth. The growth conditions were optimized for each microorganism. The complete, chemically defined medium was the Dulbecco Modified Eagle Medium (DMEM, Invitrogen, Waltham, MA, USA); casamino acids and all growth media were purchased from Difco, the amino acid solutions (Sigma, Milano, Italy) were prepared according to the amino acid concentrations present in the casamino acids. The protein hydrolysates were prepared by acid hydrolysis with 0.2 mg/mL pepsin. Tri-l-ornithine and tri-l-alanine were purchased from Bachem Inc. Bacterial growth was measured as A_620 nm_ at a fixed point after overnight growth or by following the growth kinetics with a Bioscreen Microbiology reader (Labsystem, Vantaa, Finland).

The inhibition of bacterial growth by antibiotics was also determined from the diameter of the growth inhibition halo. Bacterial suspensions obtained after overnight growth in minimal medium were diluted and inoculated in 4 mL of Agar–H_2_O (0.75%) and plated on minimal medium. Ten-microliter spots of the antibiotics were added and the growth inhibition halos measured after 24 h incubation at 37 °C.

### 4.3. Selection of Antibiotic Resistant Mutants

Antibiotic resistant mutants were isolated after growth on agar plates in the presence of 10 μg/mL GE81112 or 100 μg/mL of tri-l-ornithine. After two days incubation at 37 °C, the selected colonies were plated again in the presence of a 10-fold higher concentration of inhibitor to confirm their resistant phenotype.

### 4.4. Cell Free mRNA Translation

The 027IF2Cp(A) mRNA and the bacterial S30 cell extracts were prepared as described [38,39]. The incubation conditions for mRNA translation and for the determination of synthesized product have also been previously described [38,39].

## 5. Conclusions

An important limitation for the use of several potentially effective antibiotics is the high frequency at which resistant mutants emerge. Here we have shown that this could also be the case of GE81112, as it happens for other antibiotics, if its exclusive mechanism of entering the cells would be the active transport mediated by Opp [14]. However, the introduction of chemical modifications in this peptide molecule can allow the antibiotic to enter the cells bypassing the Opp system and would solve two problems at the same time: modified GE81112 would become effective as a broad spectrum antimicrobial agent regardless of the medium and it would reduce to <10^−9^ the frequency by which mutations of the antibiotic target that could confer resistance arise. As an alternative, GE81112 could be investigated as a narrow spectrum antibiotic effective against pathogens where the Opp transport system is essential. In any case, it can be remarked that the likelihood of the occurrence of cross-resistance phenomena should be minimized by the uniqueness of the molecular target of GE81112.

Thus, it is hoped that a derivative of this antibiotic that enters the cells via an Opp-independent route while maintaining the same molecular target could be possibly used therapeutically without resulting in a rapid selection of resistant pathogens.

## Figures and Tables

**Table 1:** Antimicrobial activity (MIC) of GE81112 on various Gram-positive and Gram-negative bacteria in growth media.

Bacteria	GE81112 MIC (μg/mL)			
	Complete Media		Minimal Media	
	Rich	Chemically Defined	Inoculum 10^4^ cfu/mL	Inoculum 10^6^ cfu/mL
*Staphylococcus aureus* Smith	>1024 ^b^			
*Staphylococcus aureus L100*	>512 ^b^	2–4	1^f^	
*Staphylococcus haemolyticus* metR ^a^	2 ^b^			
*Staphylococcus haemolyticus* metS ^a^	8 ^b^			
*Streptococcus pyogenes*	>1024			
*Streptococcus pneumoniae*	64 ^c^			
*Enterococcus faecalis* Van A ^a^	64 ^b^			
*Bacillus subtilis* ATCC6633	>1024 ^d^		0.125 ^f^	4 ^f^
*Moraxella catarrhalis* ^a^	2 ^b^			
*Haemophilus influenzae* ATCC 19418	512 ^e^			
*Escherichia coli* MG1655	>512 ^b^	2–4	0.062 ^g^	2 ^g^
*Escherichia coli MHB*	1024 ^b^			
*Pseudomonas aeruginosa* ATCC1156 ^a^	>512 ^b^		>512 ^g^	

^a^ clinical isolate; metR = methicillin resistance; metS = methicillin sensitive; VanA = vancomycin resistant; ATCC = American Type Culture Collection. Growth Media: ^b^ Mueller Hinton broth; ^c^ Todd Hewitt Broth; ^d^ antibiotic medium N°3; ^e^ Brain heart infusion + 1% supplement C; ^f^ base medium Davis Mingioli Broth + 2% glucose + 100 μg/mL asparagine; ^g^ base medium Davis Mingioli Broth + 2% glucose. Inoculum in complete media was always 10^6^ cfu/mL.

**Table 2:** Effect of proteins and protein hydrolysates on the microbiological activity (MIC) of GE81112.

Additions to Minimal Medium	GE81112 MIC (μg/mL)
None	0.030
BSA (2%)	0.125
FBS (30%)	8
BSA hydrolysate (2%)	250
Casein (2%)	0.25
Casein hydrolysate (2%)	250

Each microtiter well contained Davis Mingioli Minimal Medium; the MICs determined after 24 h incubation at 37 °C.

**Table 3:** Growth inhibition of *E. coli* strains by GE81112 and tri-l-ornithine.

*E. coli* Strain	Halo of Inhibition (mm)			
	GE81112		Orn_3_	
	1 μg	10 μg	1 μg	10 μg
SS320 (wt)	24	32	7	12
SS5012 *(ΔoppABCDF)*	0	0	0	0
SS5012 + pB2 + pBΦ30	33	43	18	26
SS5012 + pB2 (*oppBCDF*)	0	0	0	0
SS5012 + pBΦ30 (*oppA*)	0	0	0	0
SS320 (wt) + pB2 + pBΦ30	35	41	17	27

**Table 4:** Effect of genes of the *opp* operon on the sensitivity to GE81112 and tri-l-ornithine of *E. coli* DH5α.

*E. coli* Strain	Halo of Inhibition (mm)	
	GE81112 (10 μg)	Orn_3_ (10 μg)
MG1655	30	10
DH5α	0	0
DH5α + pBΦ30 (*oppA*)	32	11
DH5α + pB2 (*oppBCDF*)	27	10
DH5α + pBΦ30 + pB2	37	14

**Table 5:** Effect of 16S rRNA base substitutions on ribosome sensitivity to GE81112 and kasugamycin inhibition.

*E. coli* AVS6900916S rRNA Mutations	IC_50_ of mRNA Translation	
	GE81112 (μg/mL)	Kasugamycin (μg/mL)
wt	2	12
A794G	18	780
A794U	15	384
G926A	32	390
G926C	28	192
G926U	35	750
A1518U	3	880

**Table 6:** Bacterial strains used in this work.

Strain	Genotype	Reference
*E. coli* MG1655	LAM^−^, *rph*-1	[33]
*E. coli* MRE600	*rna*	[34,35]
*E. coli* SS320	F^−^, *lac*I22*, lac*Z*, pro-48, met*90*, trp*A*, trp*R*, his-*85*, rps*L*, azi-*9, *gyr*A*,* λ*^−^,* P1^s^	[17]
*E. coli* SS5012	Like SS320, but *rna* Δ(*trp-tdk*)	[17]
*E. coli* DH5α	Φ80d *lac*ZΔM15, *rec*A1, *end*A1, *gyr*A96, *thi*-1, *hsd*R17 (r_k_^−^, m_k_^+^), *sup*E44, *rel*A1, *deo*R, Δ(*lacZYA-argF*) U169	[36]
*B. subtilis* ATCC6633		ATCC
*B. subtilis* JH642	*trpC2, phe-1, app*A168	[37]
*B. subtilis* JH12795	*trpC2*, *phe*-1, ΔoppD, ::kan, *app*A168	[37]
*B. subtilis* JH14115	*trpC2*, *phe*-1, ::kan, (App^+^)	[37]
*B. subtilis* JH14116	*trpC2*, *phe*-1, Δ*opp*D, ::kan, (App^+^)	[37]

**Table 7:** Plasmids used in this work.

Plasmid	Vector/Insert	Reference
pBΦ30	pACY184/*E. coli, opp*A	[19]
pB2	pBR322/*E. coli, oppB*CDF	[19]

## Abbreviations

The following abbreviations are used in this manuscript:

MIC: minimal inhibitory concentration
IC_50_: concentration causing 50% inhibition
